# *Illicium verum* Extract and *Trans*-Anethole Attenuate Ovalbumin-Induced Airway Inflammation via Enhancement of Foxp3^+^ Regulatory T Cells and Inhibition of Th2 Cytokines in Mice

**DOI:** 10.1155/2017/7506808

**Published:** 2017-09-14

**Authors:** Yoon-Young Sung, Seung-Hyung Kim, Dong-Seon Kim, Ji-eun Lee, Ho Kyoung Kim

**Affiliations:** ^1^Mibyeong Research Center, Korea Institute of Oriental Medicine, 1672 Yuseong-daero, Yuseong-gu, Daejeon 305-811, Republic of Korea; ^2^Institute of Traditional Medicine and Bioscience, Daejeon University, Daejeon 300-716, Republic of Korea; ^3^KM Convergence Research Division, Korea Institute of Oriental Medicine, 1672 Yuseong-daero, Yuseong-gu, Daejeon 305-811, Republic of Korea

## Abstract

*Illicium verum* is used in traditional medicine to treat inflammation. The study investigates the effects of IVE and its component, *trans*-anethole (AET), on airway inflammation in ovalbumin- (OVA-) induced asthmatic mice. Asthma was induced in BALB/c mice by systemic sensitization to OVA, followed by intratracheal, intraperitoneal, and aerosol allergen challenges. IVE and AET were orally administered for four weeks. We investigated the effects of treatment on airway hyperresponsiveness, IgE production, pulmonary eosinophilic infiltration, immune cell phenotypes, Th2 cytokine production in bronchoalveolar lavage, Th1/Th2 cytokine production in splenocytes, forkhead box protein 3 (Foxp3) expression, and lung histology. IVE and AET ameliorated OVA-driven airway hyperresponsiveness (*p* < 0.01), pulmonary eosinophilic infiltration (*p* < 0.05), mucus hypersecretion (*p* < 0.01), and IL-4, IL-5, IL-13, and CCR3 production (*p* < 0.05), as well as IgE levels (*p* < 0.01). IVE and AET increased Foxp3 expression in lungs (*p* < 0.05). IVE and AET reduced IL-4 and increased IFN-*γ* production in the supernatant of splenocyte cultures (*p* < 0.05). Histological studies showed that IVE and AET inhibited eosinophilia and lymphocyte infiltration in lungs (*p* < 0.01). These results indicate that IVE and AET exert antiasthmatic effects through upregulation of Foxp3^+^ regulatory T cells and inhibition of Th2 cytokines, suggesting that IVE may be a potential therapeutic agent for allergic lung inflammation.

## 1. Introduction

Allergic asthma is caused by a T helper cell type 2- (Th2-) mediated immune response to common environmental allergens and is defined by chronic inflammatory lung disease. Symptoms may include recurrent episodes of wheezing, coughing, labored breathing, mucus hypersecretion secondary to airway inflammation, airway eosinophilia, bronchial hyperresponsiveness, and airway remodeling [1–3]. Multiple cell types, including mast cells, eosinophils, basophils, neutrophils, and Th2 lymphocytes, contribute to the pathogenesis of asthma by producing cytokines like interleukin- (IL-) 4, IL-5, and IL-13 [4]. Increased eosinophil and T lymphocyte numbers in bronchoalveolar lavage fluid (BALF) and bronchial mucosa are characteristic features of the inflammatory response in asthmatic patients and correlate with the severity of the disease [5]. IL-4 and IL-13 play critical roles in immunoglobulin (Ig) E isotype switching in B cells, eosinophil infiltration into lung tissues, and mucus hypersecretion [6]. IL-5 promotes the growth, differentiation, survival, and activation of eosinophils and induces the release of proinflammatory mediators and cytotoxic products [7]. In contrast, interferon- (IFN-) *γ*, which is produced by Th1 cells, regulates eosinophil recruitment in the airway by inhibiting the Th2 response [8].

*Illicium verum* Hook. f. is an aromatic evergreen tree belonging to the family *Schisandraceae*. *I. verum* fruit, commonly known as star anise, has been traditionally used in Asia to treat stomachaches, insomnia, and inflammation [9–11]. The fruits have also been used for treatment of dyspepsia, cough, and asthma [12]. *Trans*-anethole (AET) is one of the main components of *I. verum* fruit. Anethole has *in vivo* anti-inflammatory effects on animal models of nonimmune acute inflammation, including croton oil-induced ear edema and carrageenan-induced pleurisy [13]. We previously found that topical application of *I. verum* extract (IVE) improved dermatitis in house dust mite-induced NC/Nga mice by modulating the level of IgE and Th2 cytokines and chemokines [14]. AET has significant anti-inflammatory activities that are exerted by suppressing TNF-*α*/IFN-*γ*-induced expression of proinflammatory cytokines and Th2 chemokines in human keratinocytes [15]. These data suggest that *I. verum* extract may have preventive and/or therapeutic effects on various allergic diseases. However, the effect of *I. verum* and AET on allergic asthma has not yet been investigated. Thus, in this study, we investigated the anti-inflammatory effects and mechanisms of *I. verum* ethanolic extract and AET on ovalbumin- (OVA-) induced airway inflammation in an animal model of asthma.

## 2. Materials and Methods

### 2.1. Preparation of IVE

Dried fruits from *I. verum* were purchased from Omniherb Co. (Yeoungcheon, Korea), and the extract was deposited in the herbarium of the Mibyeong Research Center at the Korean Institute of Oriental Medicine (voucher specimen number: HRA-60). Dried *I. verum* plants (300 g) were extracted twice with 70% ethanol (with a 2 h reflux), and the extract was concentrated under reduced pressure. The decoction was filtered, lyophilized, and stored at 4°C. The yield of dried *I. verum* extract from starting crude materials was approximately 15.73% (wt/wt). *I. verum* extract was characterized with the two standard compounds, *p*-anisaldehyde (ChromaDex, Irvine, CA, USA) and *trans*-anethole (ChromaDex), by high-performance liquid chromatography analysis at 260 nm. IVE contained 6.14 ± 0.05 mg/g *p*-anisaldehyde and 1.98 ± 0.03 mg/g *trans*-anethole, identified at a retention time of approximately 12.4 min and 36.8 min, respectively (Supplementary Materials available online at https://doi.org/10.1155/2017/7506808) [15].

### 2.2. Animals and Materials

Seven-week-old female BALB/c mice were obtained from Orient Bio Inc. (Seongnam, Korea). Mice were housed in an air-conditioned room at a temperature of 21 ± 2°C and humidity of 50 ± 5%, under a 12 h : 12 h light : dark cycle. Mice were fed a commercial diet and water *ad libitum* for one week before beginning the experiment. The committee for animal welfare at Daejeon University approved the experimental protocols used in this study (DJUARB2014-009). All animal procedures were conducted in accordance with the guidelines of the Institutional Animal Care and Use Committee of the South Korea Research Institute of Bioscience and Biotechnology (Daejeon, Republic of Korea) and the US guidelines (NIH publication number 85-23. Revised 1996).

### 2.3. OVA Sensitization and Inhalation

The dose of IVE used in vivo model of this work was determined based on the prior experimental results of antiallergic activity of IVE. The dose-response study using OVA-induced asthmatic animal model was conducted in the doses of 50, 100, and 200 mg/kg IVE to evaluate the potential efficacy of IVE. Our findings suggest that IVE at the dose of 200 mg/kg exerts strong protective effect against Penh value to methacholine 25 mg/ml. Thus, in our present study, we evaluated and compared the antiasthmatic effects on 200 mg/kg IVE-treated mice and its constituent-treated mice.

Mice were divided into six groups with six mice per group: (1) Normal, (2) OVA-control, (3) OVA-cyclosporine A (CsA) 10 mg/kg, (4) OVA-IVE 200 mg/kg, (5) OVA-AET 20 mg/kg, and (6) OVA-AET 2 mg/kg. AET (purity: >98%) and CsA were purchased from Sigma-Aldrich Korea (Yongin, Korea). Using a modified protocol described previously [16], we mixed 50 *μ*L of 1 mg/mL A5503 albumin grade V (Sigma-Aldrich, St. Louis, MO, USA) and 80 *μ*L of 13 mg/mL A8222 aluminum hydroxide gel (Alhydrogel, Sigma-Aldrich) in 870 *μ*L of distilled water. For sensitization, all mice were immunized by intraperitoneal (ip) injection of alum-precipitated antigen (0.25 mL) containing 12.5 *μ*g of OVA (Sigma-Aldrich Korea, Seoul, Korea) and 0.26 mg of aluminum hydroxide (Sigma-Aldrich Korea) in phosphate-buffered saline (PBS) on day 0 and again day 14, followed by intratracheal injection with 2 mg of OVA (100 *μ*L) in PBS on day 3 and day 10. The mice were exposed to inhalation with 1% OVA in normal saline aerosolized using a ME-U12 ultrasonic nebulizer (Omron, Tokyo, Japan), for 30 min per day, three days per week from day 21 to day 42. On day 45, mice were exposed to inhalation with 2% OVA in normal saline. IVE (200 mg/kg) and AET (2 and 20 mg/kg) were orally administered from day 21 to day 46 on a daily basis. CsA (10 mg/kg) was orally administrated three times per week from day 21 to day 46.

**Figure 1:**
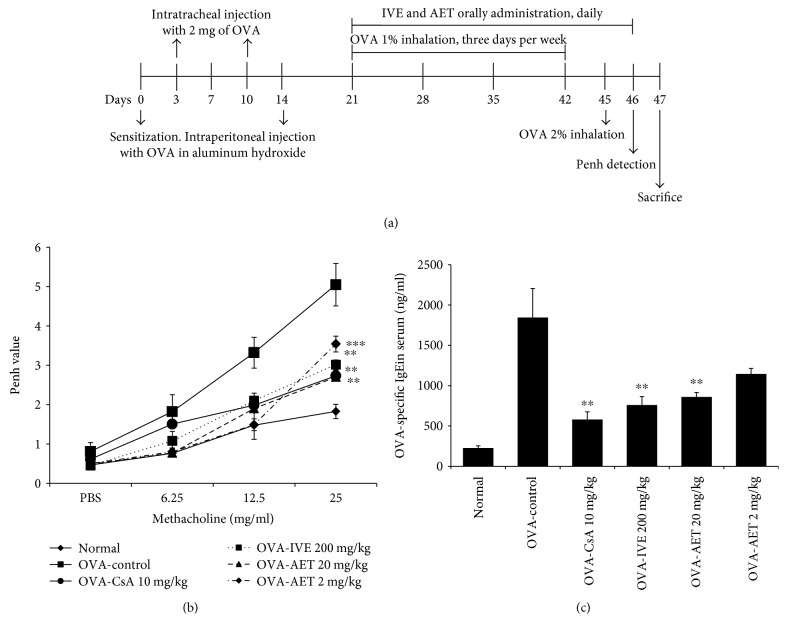
Effect of *Illicium verum* extract (IVE) and *trans*-anethole (AET) on airway hyperresponsiveness and serum IgE levels. (a) Schematic diagram of methacholine-induced airway hyperresponsiveness in mice. (b) Airway responsiveness to aerosolized methacholine (Penh) in each treatment group. (c) OVA-specific IgE levels in serum of OVA-induced mice. Results are expressed as mean ± SEM (*n* = 6 per group). ^∗∗^*p* < 0.01 and ^∗∗∗^*p* < 0.001 indicate statistically significant differences between the asthma control group and drug-treated group. Normal group: untreated Balb/c mice; OVA-Control: untreated OVA-induced asthma mice; OVA-CsA: CsA- (10 mg/kg) treated OVA mice; OVA-IVE: IVE- (200 mg/kg) treated OVA mice; OVA-AET: AET- (20 mg/kg and 2 mg/kg) treated OVA mice.

Studies using daily oral administration of CsA as an immunosuppressant have shown beneficial effects on the treatment of steroid-dependent asthma [17]. Thus, we used CsA as a positive control. One day after the last OVA exposure (2% OVA inhalation), we measured enhanced pause (Penh) to evaluate airway hyperresponsiveness. On day 47, mice were sacrificed, and serum, BALF, and lung cell samples were collected for further molecular analyses. The schematic diagram of the treatment schedule is presented in Figure 1(a).

### 2.4. Collection of BALF

Mice were sacrificed by ip injection of urethane (2.5 mg/kg) after the Penh assessment. BALF was obtained by washing the airway lamina by tracheal cannulation. By using surgical scissors, the trachea was exposed and made a small incision or punctured it with a needle to allow passage of 21 gauge lavage tube into trachea. The cannula tubing was inserted into the hole of trachea and then tied thread into single knot around it to secure the cannula in the trachea. BALF was collected by the lung lavage with 1 ml of fresh fluid *via* the trachea. After three lavages, approximately 700 *μ*L of BALF was recovered, which was centrifuged at 400 ×g for 5 min at 4°C. Lung cells were recovered by flushing 1 mL of BALF (1 mM EDTA, 10% fetal bovine serum [FBS, GIBCO, Grand Island, NY, USA], and PBS) into the lungs via the trachea. Each suspension of BALF was centrifuged, and the supernatant was collected and stored at *−*25°C for determination of cytokine levels. The cell pellets were resuspended in 1 mL PBS, and total cell number was counted using a hemocytometer. The cells (100 *μ*L) were cytospinned and stained with Diff-Quik Stain Set solution (Baxter Healthcare Corp., Miami, FL, USA) according to the manufacturer's instructions. Differential cell counts were performed based on their microscopic morphology.

### 2.5. Digestion of Pulmonary Tissue and Cell Preparations

Single cell suspensions from lung tissue and BALF were isolated using RPMI 1640 medium supplemented with 50 *μ*M 2-mercaptoethanol, 2 mM L-glutamine, 20 mM HEPES, 100 U/mL penicillin, 100 *μ*g/mL streptomycin, and 2% heat-inactivated FBS [18]. Lungs were removed from the thoracic cavity, and tissue was minced using sterile scalpels. The lung tissue was then incubated in PBS containing 1 mg/mL collagenase IV and 2 mg/mL dispase at 37°C. After incubation for 40 min, lung tissue was vigorously pipetted up and down to further dissolve remaining tissue clumps and filtered using a 70 *μ*m cell strainer (Falcon, Le Pont de Claix, France). The total cell number was determined using a hemocytometer chamber (Thermo Fisher Scientific, Grand Island, NY, USA). Between 2 × 10^3^ and 4 × 10^3^, cells were spun onto glass slides by centrifuging at 400 ×g for 4 min using a cytospin centrifuge (Cellspin, Hanil, Seoul, Korea). Differential counts were assessed according to standard morphologic criteria.

### 2.6. Flow Cytometric Analysis

Cells from lung tissue and BALF were stained with the indicated antibodies in staining solution (PBS containing 1% FBS and 0.01% NaN3) for 10 min on ice. The stained cells analyzed by two-color flow cytometry on an FACSCalibur using CellQuest software (BD Biosciences, Mountain View, Calif, USA). All antibodies such as anti-CD3, anti-CD4, anti-CD8, anti-B220, anti-CD23, anti-CD69, anti-CD11b, anti-Gr-1, and anti-CCR3 for flow cytometric analysis were purchased from BD PharMingen (San Diego, Calif, USA).

### 2.7. Determination of Penh

Airway hyperresponsiveness in mice was measured using a Buxco system (Biosystem XA; Buxco Electronics Inc., Troy, CT, USA) as previously described [16]. Mice were aerosolized with OVA for 30 min/day, three days/week for five weeks. One day after the final inhalation, mice were given aerosolized saline (0.9% NaCl), followed by increasing doses (3.15, 6.25, 12.5, 25, and 50 mg/mL) of aerosolized methacholine (Sigma-Aldrich). Airway reactivity was then monitored for 30 min, and respiratory curves were converted into Penh values. Penh is equal to Pause × PEF/PIF, where Pause = (*T*_e_ − *T*_r_)/*T*_r_ (PIF, peak inspiratory flow; PEF, peak expiratory flow; T_e_, expiratory time; T_r_, relaxation time).

### 2.8. Hematoxylin-Eosin (H&E), Masson's Trichrome (M-T), and Periodic Acid-Schiff (PAS) Staining

Lungs were removed and analyzed histologically using a modified protocol described previously [18]. Tissue was fixed in 10% (v/v) neutral-buffered formalin, embedded in paraffin, then cut into 3 *μ*m thick sections for H&E or M-T (Sigma-Aldrich Korea) staining. The degree of inflammatory cell infiltration in the airway was scored in a double-blind screen by two independent researchers. The degree of peribronchiole and perivascular inflammation was evaluated on a subjective scale of 0–2 as previously described [18]. For identification of mucus secretion in frozen lung tissue sections, 30 mm thick sections were mounted on a gelatin-coated slide and stained with PAS reagent (Sigma-Aldrich Korea). PAS-positive goblet cells were counted manually, normalized to the length of the basal bronchial epithelial perimeter, and expressed as the number of PAS-positive cells per mm of basement membrane.

### 2.9. Enzyme-Linked Immunosorbent Assay (ELISA)

The concentrations of IL-4, IL-5, and IL-13 in BALF and OVA-specific-IgE level in serum were measured using a mouse ELISA kit (R&D Systems, Minneapolis, MN, USA). OVA-specific IL-4 and IFN-*γ* production was detected in spleen cells suspended in RPMI 1640 medium supplemented with 2 mM L-glutamine and 5% FBS. Spleen cells were cultured for 48 h at a concentration of 1 × 10^5^ cells/well in 96-well plates with or without 10 *μ*g/mL of OVA in a humidified atmosphere of 5% CO_2_ at 37*°*C. The culture supernatants were collected, and IFN-*γ* and IL-4 levels were measured using an ELISA kit.

### 2.10. Forkhead Box Protein 3 (Foxp3) Immunofluorescence

Lung tissue staining was performed as previously described with some modifications [19]. Briefly, frozen blocks were randomly chosen and sectioned (5 *μ*m) with a cryostat microtome (Thermo Electron Corp., Cambridge, UK). Slides containing tissue sections were washed for 10 min in 0.1 M phosphate buffer at room temperature. Subsequently, the slides were washed twice for 10 min in 0.1 M PBS. Slides were then incubated in permeabilization buffer (0.5% Triton X-100, 0.2 *μ*g EDTA, and 1% normal donkey serum in PBS) for 30 min. Slides were rinsed twice for 10 min in PBS containing 0.1% Tween 20 (PBS-T) and blocked in 10% normal donkey serum in PBS-T for 1 h and 40 min to reduce nonspecific staining. The slides were then incubated with a rabbit antimouse Foxp3 primary antibody (1 : 1000, Abcam, Cambridge, MA, USA) diluted in blocking solution at room temperature overnight. Slides were rinsed twice for 10 min with PBS-T and incubated in H&L (Alexa Fluor® 647) conjugated donkey antirabbit IgG in blocking solution for 1 h and 20 min. DAPI was used as a nuclear stain.

### 2.11. Quantitative Reverse-Transcription Polymerase Chain Reaction (qRT-PCR)

Total RNA from the lung was extracted using RNAzol B (Tel-Test, Austin, TX, USA) according to the manufacturer's instructions. cDNA was synthesized from 3 *μ*g of total RNA using a ReverTraAce-a-cDNA Synthesis kit (Toyobo, Osaka, Japan). Proinflammatory cytokine gene expression was analyzed with SYBR Green PCR Master Mix (Applied Biosystems, Grand Island, NY, USA) and 200 nM primers. Glyceraldehyde-3-phosphate dehydrogenase (GAPDH) was used as an internal control. The TaqMan probes for Foxp3 (assay ID: Mm00475156, FAM dye-labeled) and GAPDH (part number: 4352339E, VIC dye-labeled) were selected using Assays-on-Demand Gene Expression Products (Applied Biosystems, Grand Island, NY, USA). The PCR was performed as follows: 2 min at 50°C, 10 min at 94°C, 40 cycles of 1 min at 94°C, and 1 min at 60°C. The cycle number at which the emission intensity of the sample rose above baseline was defined as the relative quantity (RQ) and was proportional to the target concentration. RT-PCR was performed in duplicate using the Applied Biosystems 7500 Fast Real-Time PCR system (Applied Biosystems) and analyzed according to the manual (threshold: 0.05, baseline: 6–15 cycles). The primer sequences were as follows: IL-5, sense 5′-GAGCACAGTGGTGAAAGAGAC-3′, antisense 5′-ATGACAGGTTTTGGAATAGCATTT-3′; IL-13, sense 5′-GCTTGCCTTGGTGGTCTTGC-3′, antisense 5′-CCATACCATGCTGCTGTTGCAC-3′; and CCR3, sense 5′-CCCGAACTGTGACTTTTGCT-3′, antisense 5′-CCTCTGGATAGCGAGGACTG-3′.

### 2.12. Statistical Analysis

Results are presented as mean *±* standard error of the mean (SEM), and differences were considered statistically significant when *p* values were less than 0.05. The values were analyzed using one-way analysis of variance (ANOVA) followed by Tukey's test for post hoc multiple comparisons (SPSS version14.0 statistic software).

## 3. Results

### 3.1. Inhibitory Effect of IVE and AET on Airway Hyperresponsiveness

To evaluate the inhibitory effect of IVE, AET, and CsA on airway hyperresponsiveness, we measured Penh in mice the first day after the final inhalation. We found that the Penh dose-response curve was shifted to the left in OVA-induced asthmatic mice, compared to normal mice (Figure 1(b)). Additionally, as shown in Figure 1(b), Penh conversion to methacholine was significantly reduced in IVE-treated (200 mg/kg), AET-treated (20 and 2 mg/kg), and CsA-treated (10 mg/kg) mice, relative to control mice sensitized with OVA.

### 3.2. Inhibition of OVA-Specific IgE in Serum

Since IgE production is closely associated with various inflammatory cells during the pathogenesis of allergic reactions [20], we examined OVA-specific IgE levels in serum of mice. OVA-specific IgE levels were markedly increased in the asthmatic control group compared with the normal group and were significantly suppressed upon treatment with IVE (200 mg/kg), AET (20 mg/kg), and CsA (10 mg/kg) (Figure 1(c)).

### 3.3. Inhibitory Effect of IVE and AET on Airway Eosinophil Accumulation and Influx of Inflammatory Cells into the Lung and BALF

**Figure 2:**
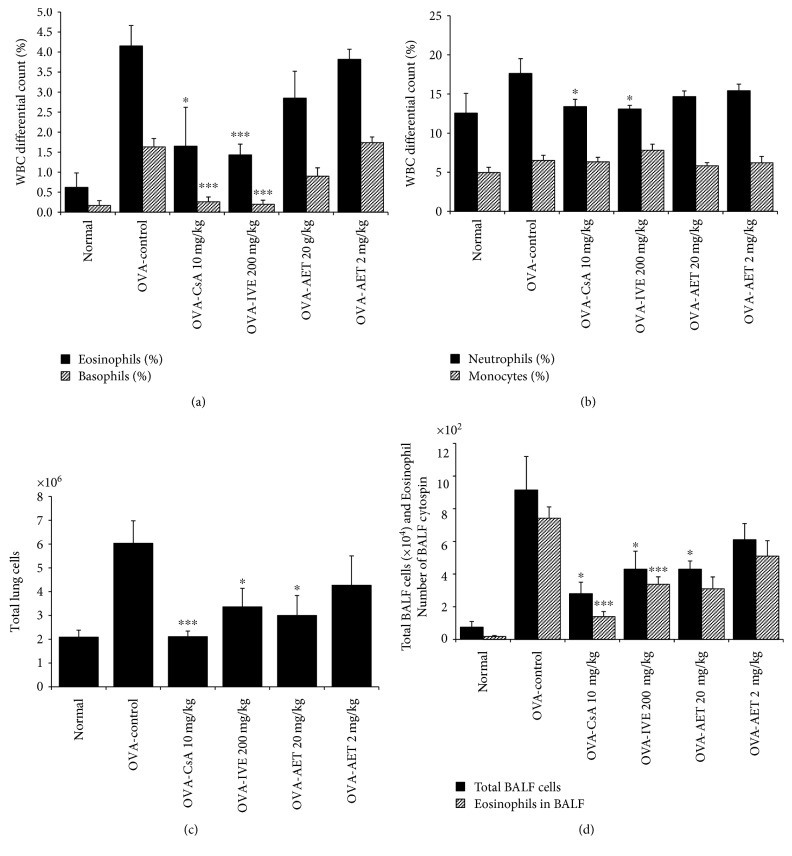
Decreased airway immune cell number and eosinophilic airway inflammation in IVE- and AET-treated OVA-induced mice. (a) Eosinophil and basophil numbers in the blood of each treatment group. (b) Neutrophil and monocyte numbers in the blood of each treatment group. (c) Total lung cells in each treatment group. (d) Total BALF cells and eosinophil number in BALF of each treatment group. Results are expressed as mean ± SEM (*n* = 6 mice per group). ^∗^*p* < 0.05 and ^∗∗∗^*p* < 0.001 indicate statistically significant differences between the asthma control group and drug-treated group. WBC: white blood cell.

Changes in the cell types present during airway inflammation in asthmatic mice, particularly eosinophils, are important course in the development and pathogenesis of asthma [20]. Eosinophils, basophils, and neutrophils were reduced in the blood of CsA- and IVE-treated mice (Figures 2(a) and 2(b)). Additionally, the total number of lung cells and eosinophils in the BALF cytospin of OVA-induced mice was higher than that that in normal mice. The total number of lung cells, as well as the number of eosinophils in BALF, was also significantly decreased in IVE- (200 mg/mg), AET- (20 mg/kg), and CsA-treated mice compared with that in control mice (Figures 2(c) and 2(d)).

### 3.4. Inhibitory Effect of IVE and AET on the Number of Immune Cell Subtypes in the Lung and BALF

To evaluate the effect of IVE and AET on immune cell subtypes, we performed flow cytometry analysis and found that the absolute numbers of CD3-, CD4-, CD8-, B220-, CD23-, CD69-, CD11b-, Gr-1-, and CCR3-positive cells in the lungs and BALF of OVA-challenged control mice were increased compared to those in saline-treated normal mice. Each cell type from IVE-, AET-, and CsA-treated mice was also significantly lower than in OVA-challenged control mice (Table 1). In particular, IVE (200 mg/kg) and CsA (10 mg/kg) treatment with OVA caused marked reduction in the numbers of CD4^+^/CD3^+^ Th cells, CD8^+^/CD3^+^ T cells, CD69^+^/CD3^+^ T cells, CD11b^+^/Gr-1^+^ granulocytes, B220^+^/CD23^+^ B cells, and CCR3^+^/CD3^−^ eosinophils in the lung. AET (20 mg/kg) treatment with OVA resulted in significant reductions in CD4^+^/CD3^+^ Th cells, CD69^+^/CD3^+^ T cells, CD11b^+^/Gr-1^+^ granulocytes, B220^+^/CD23^+^ B cells, and CCR3^+^/CD3^−^ eosinophils. Finally, IVE (200 mg/kg), AET (20 mg/kg), and CsA (10 mg/kg) treatment with OVA also significantly decreased the number of CD4^+^/CD3^+^ Th cells, CD11b^+^/Gr-1^+^ granulocytes, and CCR3^+^/CD3^−^ eosinophils in BALF. The deficits in CCR3^+^/CD3^−^ eosinophils in the lung and BALF were accompanied by decreased eosinophils in the BALF cytospin (Figure 2(d)).

### 3.5. Histological Analysis of Lung Tissue

**Figure 3:**
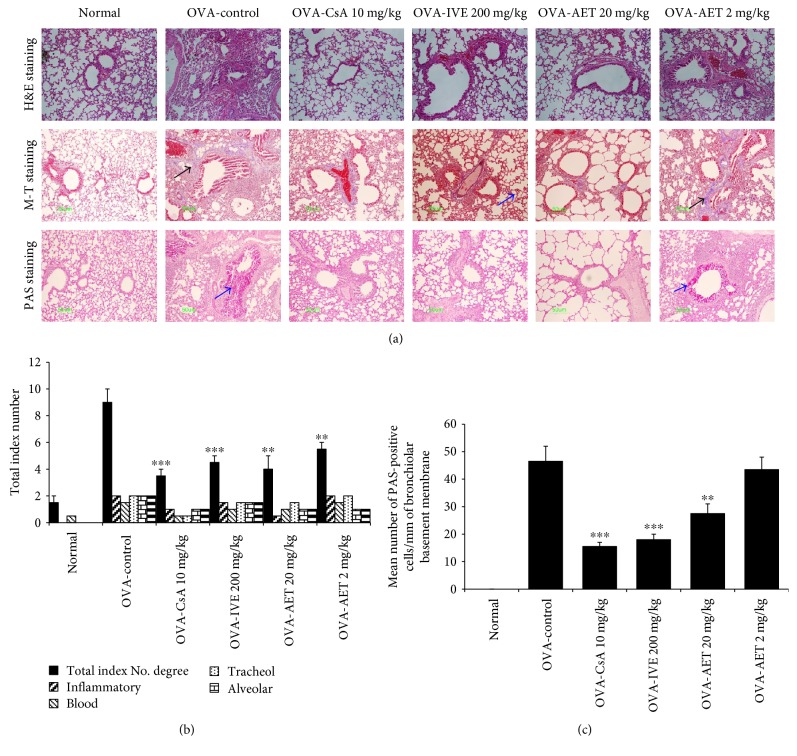
Effect of *Illicium verum* extract (IVE) and *trans*-anethole (AET) on histologic markers of airway inflammation in lung tissue of OVA-induced mice. (a) H&E, M-T, and PAS staining of lung sections from normal and asthmatic mice treated with OVA (control), IVE (200 ng/kg), or AET (20 or 2 mg/kg). Total index number (b) and PAS-positive cell number (c) were quantitated. H&E: Hematoxylin-eosin; M-T: Masson's trichrome; PAS: periodic acid-Schiff. Results are expressed as mean ± SEM (*n* = 6 mice per group). ^∗∗^*p* < 0.01 and ^∗∗∗^*p* < 0.001 indicate statistically significant differences between the asthma control group and drug-treated group.

Histological investigation of lung tissue sections from OVA-challenged control mice revealed inflammatory changes, including peribronchial and perivascular infiltration of inflammatory cells, including eosinophils, mast cells, and lymphocytes. In contrast, histological sections from IVE-, AET-, and CsA-treated mice showed reduced airway inflammation, characterized by decreased inflammatory cell infiltrate and fibrosis (Figures 3(a) and 3(b)). The degree of mucus overproduction caused by goblet cell hyperplasia within the bronchi was evaluated by PAS staining. The lung tissue of OVA-challenged control mice had significantly increased mean numbers of PAS-positive cells compared with normal mice. Additionally, IVE, AET, and CsA treatments significantly reduced the mean number of PAS-stained goblet cells (Figures 3(a) and 3(c)). Together, these results indicate that IVE and AET inhibit pathological changes in lung tissue of asthmatic mice.

### 3.6. Effect of IVE and AET on mRNA Expression of Foxp3, IL-5, IL-13, and CCR3 in Lung Tissue

**Figure 4:**
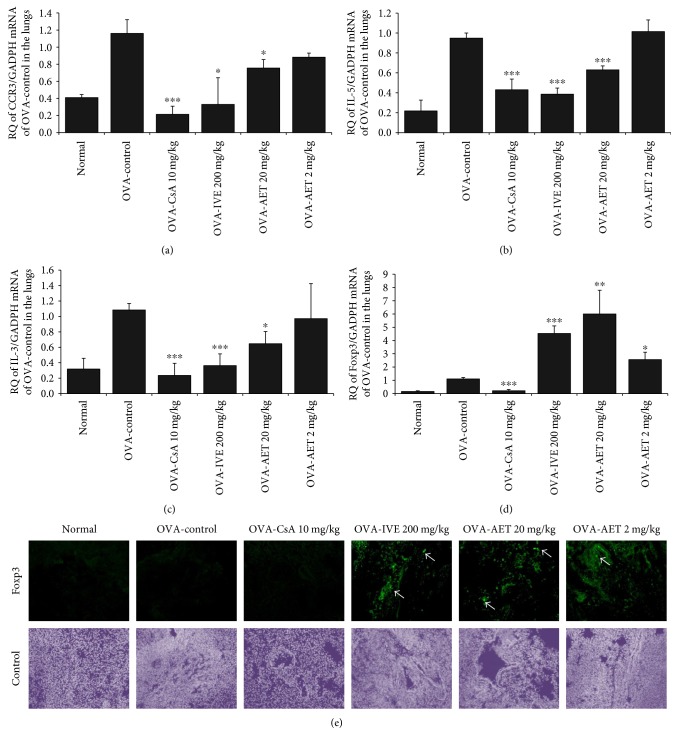
Effect of IVE and AET on mRNA expression of Foxp3, IL-5, IL-13, and CCR3 and immunofluorescence staining for Foxp3 in lung tissue of OVA-induced asthmatic mice. (a) CCR3, (b) IL-5, (c) IL-13, and (d) Foxp3 mRNA expression. (e) Immunofluorescent staining for Foxp3 (green) and DAPI (DNA) (gray) in lung tissue. Results are expressed as mean ± SEM (*n* = 6 mice per group). ^∗^*p* < 0.05, ^∗∗^*p* < 0.01, and ^∗∗∗^*p* < 0.001 indicate statistically significant differences between the asthma control group and drug-treated group.

**Figure 5:**
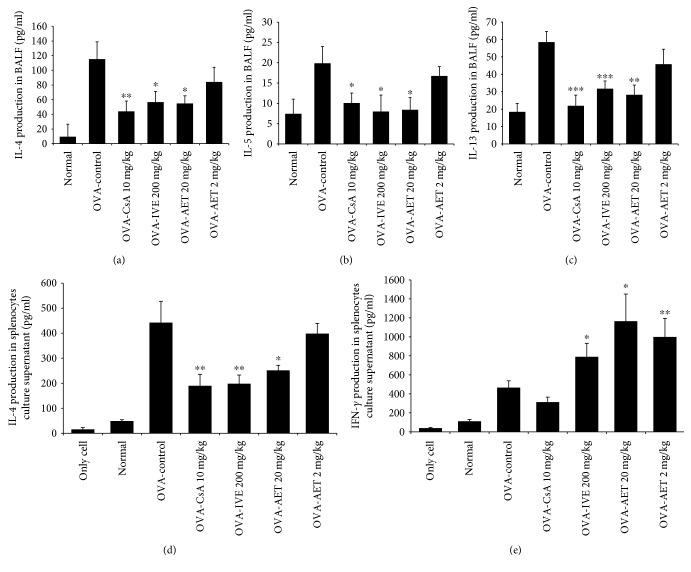
Effect of IVE and AET on cytokine production in BALF and splenocytes. BALF production of (a) IL-4, (b) IL-5, and (c) IL-13. Splenocyte production of (d) IL-4 and (e) IFN-*γ*. Results are expressed as mean ± SEM (*n* = 6 mice per group). ^∗^*p* < 0.05, ^∗∗^*p* < 0.01, and ^∗∗∗^*p* < 0.001 indicate statistically significant differences between the asthma control group and drug-treated group.

As shown in Figures 4(a), 4(b), and 4(c), CCR3, IL-5, and IL-13 transcript expression was increased in the lung tissue of OVA-challenged control mice; IVE (200 mg/kg), AET (20 mg/kg), and CsA (10 mg/kg) treatments significantly reduced transcript expression of these targets compared with control mice. This decrease was accompanied by changes in eosinophil influx (CCR3^+^/CD3^−^ cells, Table 1) and BALF cytokines (IL-5 and IL-13, Figures 5(b) and 5(c)). However, Foxp3, a major transcription factor that controls regulatory T cell function in allergic inflammation [21], transcript, and protein expression in the lung were increased in IVE- (200 mg/kg) and AET-treated (20 and 2 mg/kg) mice (Figures 4(d) and 4(e)).

### 3.7. Effect of IVE and AET on Cytokine Production in BALF and Splenocyte Culture Supernatants

To determine whether IVE and AET influence cytokine secretion in BALF and splenocytes, we measured IL-4, IL-5, IL-13, IL-4, and IFN-*γ* levels by ELISA after the final OVA challenge. As shown in Figures 5(a), 5(b), and 5(c), IL-4, IL-5, and IL-13 levels were significantly reduced in BALF of IVE- (200 mg/kg), AET- (20 mg/kg), and CsA-treated (10 mg/kg) mice. Additionally, we found that IVE and AET inhibited IL-4 (Th2 cytokine) production and increased IFN-*γ* (Th1 cytokine) production in splenocytes (Figures 5(d) and 5(e)).

## 4. Discussion

Allergic diseases, such as asthma and atopic dermatitis, are chronic inflammatory diseases mediated by Th2-driven inflammation in response to common environmental allergens and are increasing in prevalence [22]. Our previous studies reported that topical application of *I. verum* ethanolic extract in mite antigen-induced atopic dermatitis mice showed remarkable antiallergic properties exerted through inhibition of Th2 cell responses [14]. Therefore, we hypothesized that IVE may exhibit therapeutic effects on allergic asthma.

Th2 lymphocytes play a critical role in the initiation and progression of allergic diseases, including atopic dermatitis and asthma, by releasing IL-4, IL-5, and IL-13 [23]. The production of these cytokines contributes to IgE production and inflammatory responses, such as airway infiltration, eosinophil activation, mast cell differentiation, and mucus hypersecretion [22, 24]; infiltrating eosinophils in the airway induce mucus secretion by epithelial goblet cells [25]. We found that IVE and AET treatment in OVA-induced mice significantly reduced B cell-dependent production of OVA-specific IgE in serum, which was associated with decreased numbers of B220^+^/CD23^+^-activated B cells in the lung and BALF. IVE and AET treatment also decreased IL-4 and IL-13 mRNA expression in lung tissue, as well as eosinophilia and IL-4, IL-5, and IL-13 production in BALF. Our findings suggest that the inhibitory effect of IVE and AET on eosinophilia and levels of Th2 cytokines attenuates mucus hypersecretion.

Eosinophil infiltration and peripheral eosinophilia are dependent on the eosinophil-specific cytokine (IL-5) and chemokines (eotaxin and RANTES), which provoke an eosinophilic response in the peripheral blood and airways via the C-C chemokine receptor type 3 (CCR3) [26]. CCR3 mRNA and protein levels are elevated in the bronchial mucosa of asthmatics, and this enhanced expression is associated with airway hyperresponsiveness [27]. Here, IVE and AET treatment reduced CCR3 mRNA expression in lung tissue, which correlated with decreased CD3^−^/CCR3^+^ eosinophils in the lung and BALF. One of the immunologic features of asthma is a shift of T helper (Th) 1/Th2 balance toward Th2 [28]. Th1 cells antagonize the effects of Th2 cells, and thus, increase of the balance of Th1/Th2 is one goal for treatment of allergic asthma [29]. Therefore, in the study, the effect of IVE and AET on Th1/Th2 balance was studied by assessment of IL-4 and IFN-*γ* gene expression in splenocytes. Supporting this, IVE and AET treatment led to increased Th1 cytokine (IFN-*γ*) gene expression and decreased Th2 cytokine (IL-4) gene expression in splenocytes of OVA-induced mice.

Foxp3 is a key transcription factor that controls regulatory T cell function [21], and the absence of Foxp3 is associated with development of severe allergic inflammation and elevated IgE levels in mice and humans [30]. CD4^+^/CD25^+^ regulatory T cells suppress pathogenic Th2 responses in Th2-mediated allergic inflammatory diseases; thus, induction of allergen-specific regulatory T cells has recently been considered to be an attractive strategy for asthma therapy [31]. In our current study, mRNA and protein expression of Foxp3 was increased in the lungs of IVE- and AET-treated mice, but CsA had no significant effect on Foxp3 expression. Moreover, AET treatment significantly enhanced expression of Foxp3 mRNA in a dose-dependent manner in the lung. Together, these findings suggest that IVE and AET may suppress Th2 cytokine production by modulating the activity of regulatory T cells.

From the previous HPLC analysis, it was showed that IVE contains anisaldehyde and *trans*-anethole as main constituents [15]. In this work, 2 mg/kg of trans-anethole (AET) effectively did not inhibit the lung inflammation, while at 10 times concentration, AET showed a similar inhibitory effect like the whole extract (IVE). Anisaldehyde at the same concentrations did not show the inhibitory effect on airway hyperresponsiveness (data not shown). These results suggest that *trans*-anethole may be partly responsible for the anti-inflammatory activity of IVE. However, we did not exclude the possibility that the effect of IVE is a combination of effects from more than one constituents. Thus, further study is needed to definitively determine the antiallergic components in IVE.

## 5. Conclusions

IVE and AET suppress airway hyperresponsiveness, eosinophil infiltration, serum IgE, and Th2 cytokine production in an OVA-challenged mouse asthma model. Importantly, IVE and AET significantly increase Th1 cytokine production and Foxp3 expression. Thus, these findings suggest that IVE and AET may be potent immune-regulatory agents for the prevention and/or treatment of allergic lung inflammation that act by enhancing Foxp3^+^ regulatory T cells and inhibiting Th2 cytokines.

## Supplementary Material

Figure: HPLC chromatograms of (A) standard mixture and (B) 70% ethanol extract of Illicium verum at 260 nm. (1) p-Anisaldehyde; (2) trans-anethole. IVE contained 6.14±0.05 mg/g anisaldehyde and 1.98±0.03 mg/g anethole, identified at a retention time of approximately 12.4 min and 36.8 min, respectively (Sung et al., 2012).

## Figures and Tables

**Table 1:** Quantification of immune cell subtypes in lungs and BALF by FACS analysis.

Cell phenotypes in lungs and BALF		Normal Balb/c mice	OVA-induced asthma mice (total absolute number)				
			Control	CsA (10 mg/kg)	IVE (200 mg/kg)	AET (20 mg/kg)	AET (2 mg/kg)
Lung	CD4^+^/CD3^+^ (1 × 10^6^ cells)	2.6 ± 0.77	9.6 ± 0.18	3.6 ± 0.44^∗∗∗^	3.2 ± 0.32^∗∗∗^	4.9 ± 0.29^∗∗∗^	8.3 ± 0.23^∗∗∗^
	B220^+^/CD23^+^ (1 × 10^6^ cells)	0.5 ± 0.09	7.1 ± 0.77	1.2 ± 0.18^∗∗∗^	1.4 ± 0.02^∗∗∗^	2.6 ± 0.23^∗∗∗^	4.8 ± 0.34^∗∗^
	CD8^+^/CD3^+^ (1 × 10^6^ cells)	1.7 ± 0.43	4.1 ± 1.14	1.9 ± 0.09^∗^	1.7 ± 0.19^∗^	2.3 ± 0.04	3.5 ± 0.21
	CD69^+^/CD3^+^ (1 × 10^5^ cells)	0.7 ± 0.06	7.3 ± 0.23	2.7 ± 0.20^∗∗∗^	2.7 ± 0.15^∗∗∗^	4.0 ± 0.52^∗∗∗^	4.7 ± 0.29^∗∗∗^
	CD11b^+^/Gr-1^+^ (1 × 10^6^ cells)	0.6 ± 0.00	7.2 ± 0.55	2.3 ± 0.30^∗∗∗^	2.7 ± 0.18^∗∗∗^	3.9 ± 0.14^∗∗∗^	4.5 ± 0.74^∗∗^
	CCR3^+^/CD3^−^ (1 × 10^5^ cells)	0.7 ± 0.02	13.5 ± 1.05	3.3 ± 0.46^∗∗∗^	3.9 ± 0.05^∗∗∗^	6.0 ± 0.01^∗∗∗^	8.1 ± 2.72^∗^

BALF	CD4^+^/CD3^+^ (1 × 10^6^ cells)	0.5 ± 0.29	30.5 ± 5.20	6.4 ± 1.93^∗∗∗^	6.6 ± 1.86^∗∗∗^	7.8 ± 0.03^∗∗∗^	30.6 ± 3.81
	CD11b^+^/Gr-1^+^ (1 × 10^6^ cells)	0.4 ± 0.29	15.1 ± 4.03	3.4 ± 0.48^∗∗^	4.6 ± 0.66^∗^	3.8 ± 1.03^∗∗^	8.2 ± 2.53
	CCR3^+^/CD3^−^ (1 × 10^5^ cells)	0.2 ± 0.09	13.5 ± 1.42	2.0 ± 0.41^∗∗∗^	4.5 ± 1.28^∗∗∗^	3.8 ± 0.39^∗∗∗^	9.9 ± 0.13^∗^

Results are expressed as mean ± SEM (*n* = 6 mice per group). ^∗^*p* < 0.05, ^∗∗^*p* < 0.01, and ^∗∗∗^*p* < 0.001 indicate statistically significant differences between the asthma control group and drug-treated group.

## Abbreviations

AET: *Trans*-anethole
BALF: Bronchoalveolar lavage fluid
CCR3: C-C chemokine receptor type 3
CsA: Cyclosporine A
Foxp3: Forkhead box protein 3
IgE: Immunoglobulin E
IL: Interleukin
IVE: *Illicium verum* extract
OVA: Ovalbumin
Penh: Enhanced pause
Th: T helper.
