# Supplementary material for: Illicium verum Extract and Trans-Anethole Attenuate Ovalbumin-Induced Airway Inflammation via Enhancement of Foxp3+ Regulatory T Cells and Inhibition of Th2 Cytokines in Mice
Source: Mediators Inflamm. 2017 Sep 14;2017:7506808. doi: 10.1155/2017/7506808 (PMC5618762; doi:10.1155/2017/7506808)
Supplement: Supplementary file 1 — Figure: HPLC chromatograms of (A) standard mixture and (B) 70% ethanol extract of Illicium verum at 260 nm. (1) p-Anisaldehyde; (2) trans-anethole. IVE contained 6.14±0.05 mg/g anisaldehyde and 1.98±0.03 mg/g anethole, identified at a retention time of approximately 12.4 min and 36.8 min, respectively (Sung et al., 2012). [file 7506808.f1.pptx]

## Slide 1
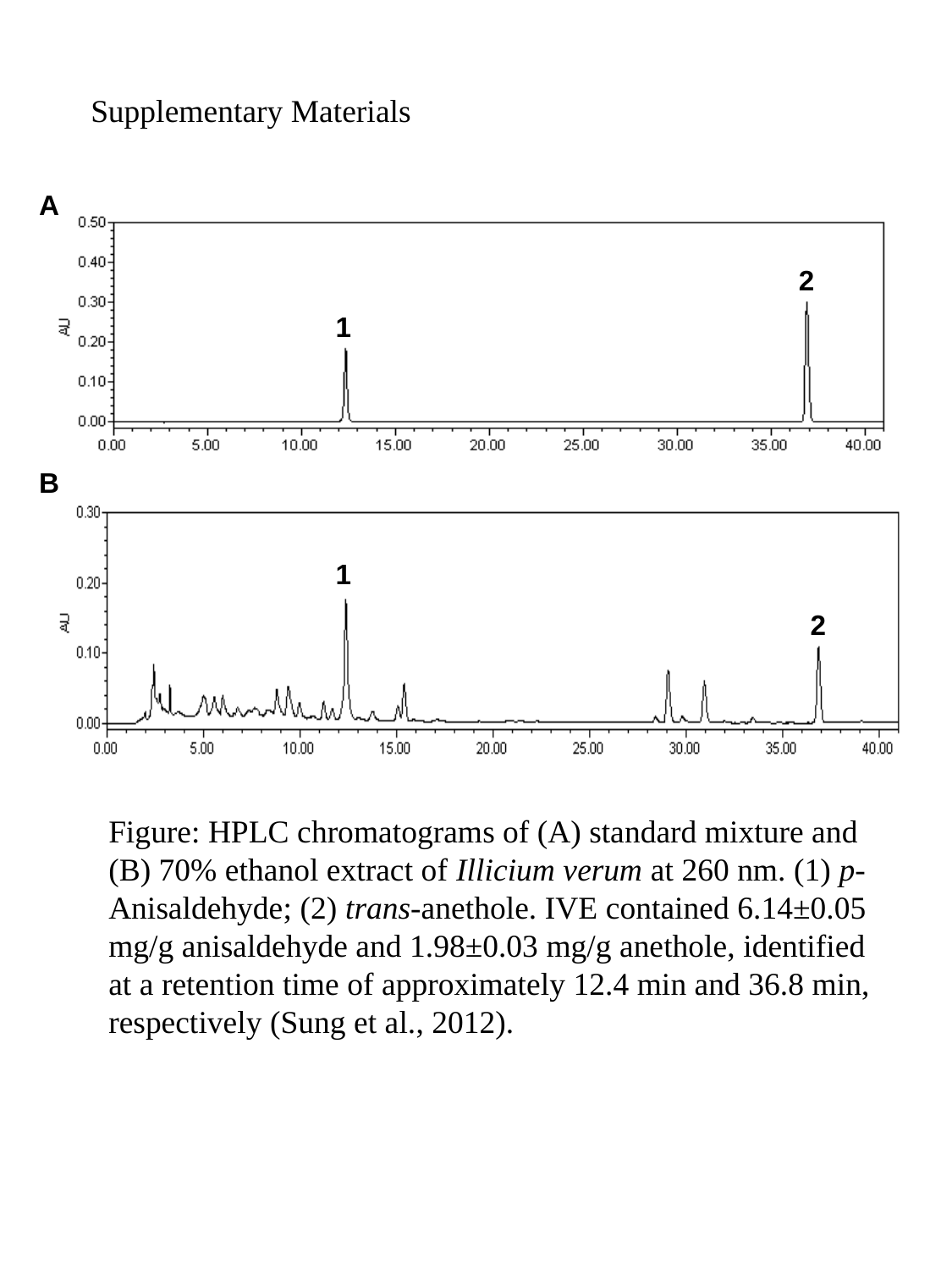

Supplementary Materials
A
2
1
B
1
2
Figure: HPLC chromatograms of (A) standard mixture and (B) 70% ethanol extract of Illicium verum at 260 nm. (1) p-Anisaldehyde; (2) trans-anethole. IVE contained 6.14±0.05 mg/g anisaldehyde and 1.98±0.03 mg/g anethole, identified at a retention time of approximately 12.4 min and 36.8 min, respectively (Sung et al., 2012).
